# Distribution of Virulence Determinants (esp, cylA, and gelE) Among Clinical Enterococcus Isolates: A Molecular Study From Central India

**DOI:** 10.7759/cureus.110174

**Published:** 2026-06-03

**Authors:** Bhawani Shankar Verma, Prashanth K Guddeti, Ramanath Karicheri, Md Abdullah, Hemant B Patil

**Affiliations:** 1 Microbiology, Prince Medical College and Hospital, Sikar, IND; 2 Microbiology, Dr. Ulhas Patil Medical College and Hospital, Jalgaon, IND; 3 Microbiology, Index Medical College Hospital and Research Center, Indore, IND; 4 Biotechnology, Jain Irrigation Systems Pvt Ltd., Jalgaon, IND

**Keywords:** cytolysin (cyla), enterococcal infection, enterococcal surface protein (esp), enterococcus species, gram-positive cocci

## Abstract

Background: The genus Enterococcus contains gram-positive cocci. Before the widespread use of antibiotics, enterococci were known causes of endocarditis, urinary tract infections (UTIs), and intra-abdominal infections. The three most important enterococcal virulence agents discovered are cytolysin (cylA), enterococcal surface protein (esp), and gelatinase (gelE). Serious concerns about infectious illnesses are raised by the increasing number of genes for antibiotic resistance.

Materials and methods: The study was granted permission by the Institutional Ethical Committee (MU/Research/EC/Ph.D./2021/58), and every single sample was taken from patients who were hospitalized at the Index Medical College Hospital and Research Center in Indore, India. One hundred and six enterococcal isolates were collected between December 2021 and August 2024, and their species distribution was examined. Standard molecular techniques were used to identify virulence genes, such as cytolysin (cylA), enterococcal surface protein (esp), and gelatinase (gelE).

Results: Among 106 Enterococcus isolates analyzed by reverse transcription polymerase chain reaction (RT-PCR), the esp gene was the most prevalent (74.53%), followed by cylA (55.66%) and gelE (45.28%). *Enterococcus faecalis* showed higher frequencies of virulence genes compared to *Enterococcus avium*. High resistance was observed to erythromycin, penicillin, chloramphenicol, and tetracycline, whereas most isolates remained sensitive to linezolid and vancomycin. These findings indicate the presence of virulence-associated genes and variable antimicrobial resistance patterns among the Enterococcus isolates.

Conclusion: The study reveals a significant pathogenic potential of Enterococcus strains, evidenced by the high prevalence of major virulence genes (esp, cylA, and gelE).

## Introduction

Enterococcus was primarily reported in the year 1899 as a new Streptococcus of gut organism. The same cause of identical gram-positive cocci was found in a patient with endocarditis in 1906 [1]. The genus Enterococcus contains gram-positive cocci that can be found individually, in pairs, or in short chains. When Gram stains are made from solid media growth, cells might become coccobacillus. When preparing Gram stains from thioglycolate broth, cells tend to be ovoid and in chains [2,3]. Prior to the widespread use of antibiotics, enterococci were known causes of endocarditis, urinary tract infections (UTIs), and intra-abdominal infections. The widespread use of antimicrobial drugs was associated with increased acknowledgment of the problem of enterococcal infection and an increase in the prevalence of enterococci in hospital-acquired illnesses [4,5]. Furthermore, the expansion of genes for antibiotic resistance raises serious concerns regarding infectious diseases. Cytolysin (cylA), enterococcal surface protein (esp), and gelatinase (gelE) are the most significant enterococcal infectious agents that have been found [6]. Certain virulence factors, including the genes for agg, esp, and cylA, have been discovered to be contained on a 153-kb pathogenicity island [7]. The virulence factors of enterococci may be linked to their persistence in a hospital environment [8]. Determining whether the resistance in the genetic elements is effectively transferred or if the isolates' resistance increased in a clonal is crucial. The potential of controlling a microbe that can both effectively disperse and effectively transfer resistance represents the worst-case scenario. Enterococcus is the best illustration of the latter phenomenon, which has become a major nosocomial infection within the past ten years [9-11].

## Materials and methods

The study was granted permission by the Institutional Ethical Committee (MU/Research/EC/Ph.D./2021/58), and all samples were collected from patients hospitalized at the Index Medical College Hospital and Research Center, Indore, India. A total of 106 Enterococcal isolates were collected between December 2021 and August 2024, and their species distribution was analyzed. 

Inclusion criteria 

Clinical isolates of Enterococcus species obtained during the study period were included. These isolates were recovered from various clinical specimens, such as blood, urine, pus, aspirated fluids, cerebrospinal fluid (CSF), and other body fluids collected from admitted patients. Only those patients who provided written informed consent for participation in the study were included. Relevant clinical and demographic details were recorded using a standardized patient proforma.

Exclusion criteria

Isolates of Enterococcus species obtained from patients who did not provide consent to participate in the study were excluded. Additionally, duplicate isolates from the same patient and sputum and stool samples, and incomplete clinical information or inadequate quality for analysis were excluded.

*Enterococci* strains were obtained from a variety of clinical specimens submitted to the microbiology laboratory, including blood, urine, exudates (surgical and non-surgical wound swabs and pus), catheters, ascitic fluid, synovial fluid, pleural fluid, and other body fluids. Depending on the specimen type, samples were cultured on appropriate media, including Trypticase Soy Agar with 5% sheep blood, MacConkey agar, and Bile Esculin Azide agar (HiMedia Laboratories Pvt. Ltd., Mumbai, India), followed by incubation at 37°C overnight [12]. Antibiotic susceptibility testing was performed using the Kirby-Bauer disk diffusion method on Mueller-Hinton agar and Mueller-Hinton agar supplemented with 5% sheep or human blood (HiMedia Laboratories Pvt. Ltd., Mumbai, India). The antibiotics tested included penicillin (10 units), chloramphenicol (30 µg), erythromycin (15 µg), tetracycline (30 µg), doxycycline (30 µg), ciprofloxacin (5 µg), high-level gentamicin (120 µg), vancomycin (30 µg), linezolid (15 µg), nitrofurantoin (300 µg), and norfloxacin (for urinary isolates only) (HiMedia Laboratories Pvt. Ltd., Mumbai, India). Interpretation of results was performed according to Clinical and Laboratory Standards Institute (CLSI) guidelines (2022-2023) [12].

Molecular detection of virulence genes by real-time polymerase chain reaction (PCR)

Genomic DNA was extracted using the HiPurA® Bacterial Genomic DNA Purification Kit (MB505) (HiMedia Laboratories Pvt. Ltd., Mumbai, India) according to the manufacturer’s protocol. The extracted DNA was used for the detection of target genes (esp, cylA, gelE) using real-time PCR. The HiPurA® kit provides a rapid and efficient method for isolating high-quality genomic DNA suitable for molecular applications. It employs a silica-based HiElute Miniprep Spin Column system for DNA purification. Cell lysis is achieved using lysozyme and Proteinase K, followed by purification through centrifugation. DNA quality and quantity were assessed using agarose gel electrophoresis and spectrophotometric analysis.

Binding step

Two hundred microliters of ethanol (95-100%) was added to the lysate, mixed, and transferred to the HiElute Miniprep Spin Column (HiMedia Laboratories Pvt. Ltd., Mumbai, India) [13].

Prewash step

Five hundred microliters of Prewash Solution and Wash Solution (HiMedia Laboratories Pvt. Ltd., Mumbai, India) were added sequentially, and the mixture was centrifuged as per protocol [13].

Elution step

DNA was eluted using 200 µl Elution Buffer (HiMedia Laboratories Pvt. Ltd., Mumbai, India) [13].

Real-time polymerase chain reaction (PCR) conditions 

Amplification was carried out using the Bio-Rad CFX96 Real-Time PCR System (Bio-Rad Laboratories, Hercules, CA, USA). The cycling conditions included initial denaturation at 95°C for 15 minutes, followed by 30 cycles of denaturation at 94°C for 1 minute, annealing at 56°C for 1 minute, extension at 72°C for 1 minute, and final extension at 72°C for 10 minutes [13].

Statistical analysis 

Data were compiled and analyzed using Microsoft Excel (Microsoft Corporation, Redmond, WA, USA) and Minitab 17 (Minitab LLC, State College, PA, USA). Descriptive statistics and Chi-square test were applied, with significance set at p < 0.05.

## Results

The distribution of virulence genes among *Enterococcus* isolates is depicted. A total of 106 isolates were analyzed for the presence of virulence genes using real-time PCR. Out of 106 isolates, 79 (74.53%) were positive for the esp gene, while 27 (25.47%) were negative. For the cylA gene, 59 (55.66%) isolates were positive, and 47 (44.34%) were negative. Similarly, 48 (45.28%) isolates were positive for the gelE gene, whereas 58 (54.72%) were negative. Overall, the esp gene was the most prevalent virulence factor, followed by cylA and gelE genes among the studied *Enterococcus* isolates (Table 1).

**Table 1:** Association of virulence genes with gender among Enterococcus isolates. Data are presented as frequency (percentage). The association between gender and virulence genes (esp, cylA, and gelE) was analyzed using the chi-square test. Pairwise chi-square analysis demonstrated that esp (χ² = 23.68, p < 0.001) and gelE (χ² = 9.93, p = 0.002) genes showed strong significant associations, while the cylA gene showed a modest but statistically significant association (χ² = 4.31, p = 0.038).

Gene	Group	Overall n (%)	Chi-square	p-value
esp gene	Positive	79 (74.53%)	23.68	0.0001
	Negative	27 (25.47%)		
cylA gene	Positive	59 (55.66%)	4.31	0.038
	Negative	47 (44.34%)		
gelE gene	Positive	50 (47.17%)	9.93	0.002
	Negative	56 (52.83%)		

The distribution of virulence genes among *Enterococcus *species across various clinical samples is depicted. All 106 *Enterococcus* isolates, the distribution of virulence genes among *Enterococcus* isolates was analyzed according to gender. A total of 106 isolates were included, comprising 50 males (47.16%) and 56 females (52.83%). The presence of *esp,* *cylA,* and *gelE* genes was determined using RT-PCR. Among male patients (n = 50), 39 (78%) isolates were positive for the esp gene, 24 (48%) for the cylA gene, and 25 (50%) for the gelE gene. The corresponding negative isolates were 11 (22%), 26 (52%), and 25 (50%), respectively. Among female patients (n = 56), 40 (71.43%) isolates were positive for the esp gene, 35 (62.50%) for the cylA gene, and 25 (44.65%) for the gelE gene. The negative isolates were 16 (28.57%), 21 (37.50%), and 31 (55.35%), respectively (Table 2). 

**Table 2:** Table shows the total percentage of virulence genes between gender wise in the Enterococcus species. Values are expressed as n (%). Percentages are column-wise. P-values were calculated using the chi-square test where appropriate. A p-value <0.05 was considered significant. Statistical analysis using the chi-square test showed no significant association between gender and the presence of virulence genes (esp: χ² = 0.60, p = 0.44; cylA: χ² = 2.25, p = 0.13; gelE: χ² = 0.85, p = 0.36), indicating that the distribution of these genes was not significantly different between male and female patients (p > 0.05).

Genes	Groups	Gender		Chi-square	p-value
		Male 50 (47.16%)	Female 56 (52.83%)		
esp gene	Positive	39 (78%)	40 (71.43%)	0.60	0.44
	Negative	11 (22%)	16 (28.57%)		
cylA gene	Positive	24 (48%)	35 (62.50%)	2.25	0.13
	Negative	26 (52%)	21 (37.50%)		
gelE gene	Positive	25 (50%)	25 (44.65%)	0.85	0.36
	Negative	25 (50%)	31 (55.35%)		

The association between the esp gene and different clinical samples among Enterococcus infection isolates was analyzed using a total of 106 samples. The distribution of samples included urine (n = 77), blood (n = 12), pus (n = 11), body fluids (n = 4), and swab samples (n = 2). Out of the total isolates, 79 (74.53%) were positive for the esp gene, while 27 (25.47%) were negative. Among urine samples, 60 (75.94%) were esp gene positive and 17 (62.96%) were negative. In blood samples, 8 (10.13%) isolates were positive and 4 (14.81%) were negative. Similarly, pus samples showed 8 (10.13%) positive and 3 (11.11%) negative isolates. Body fluid samples demonstrated 2 (2.53%) positive and 2 (7.41%) negative isolates, whereas swab samples showed 1 (1.26%) positive and 1 (3.70%) negative isolate (Table 3).

**Table 3:** Distribution of the esp gene among different clinical sample types. Values are expressed as n (%). Percentages are column-wise. P-values were calculated using the chi-square test where appropriate. A p-value <0.05 was considered significant. Overall, although higher esp gene positivity was observed in pus, body fluids, and swab samples, the association between the esp gene and different clinical samples was not statistically significant (χ² = 2.78, p = 0.597; p > 0.05).

Sample	esp Gene positive n=79 (74.53%)	esp Gene negative n=27 (25.47%)	Chi-square	P-value
Urine (77)	60 (75.94%)	17 (62.96%)	2.78	0.597
Blood (12)	8 (10.13%)	4 (14.81%)		
Pus (11)	8 (10.13%)	3 (11.11%)		
Body fluids (04)	2 (2.53%)	2 (7.41%)		
Swab (02)	1 (1.26%)	1 (3.70%)		

The association between the cylA gene and different clinical sample types among Enterococcus isolates was analyzed using a total of 106 isolates. The samples included urine (n = 77), blood (n = 12), pus (n = 11), body fluids (n = 4), and swab samples (n = 2). Among these, urine samples showed 41 isolates (69.49%) positive for the cylA gene, while 36 (76.60%) were negative. In blood samples, 5 isolates (8.47%) were positive, whereas 7 (14.89%) were negative. Pus samples demonstrated 7 isolates (11.86%) positive and 4 (8.51%) negative. All isolates from body fluids showed 4 (6.78%) positive and none (0%) negative. Similarly, swab samples showed 2 isolates (3.39%) positive, with no negative isolates (0%) observed. Overall, 59 (55.66%) isolates were positive for the cylA gene, while 47 (44.34%) were negative (Table 4).

**Table 4:** Distribution of the cylA gene among different clinical sample types. Values are expressed as n (%). Percentages are column-wise. P-values were calculated using the chi-square test where appropriate. A p-value <0.05 was considered significant. Overall, although higher cylA gene positivity was observed in pus, body fluids, and swab samples, the association between esp gene presence and type of clinical sample was statistically not significant (chi-square = 6.20, p = 0.185; p > 0.05).

Sample	cylA gene positive n=59 (55.66%)	cylA gene negative n=47 (44.34%)	Chi-square	P-value
Urine (77)	41 (69.49%)	36 (76.60%)	6.20	0.185
Blood (12)	5 (8.47%)	7 (14.89%)		
Pus (11)	7 (11.86%)	4 (8.51%)		
Body fluids (04)	4 (6.78%)	0 (0%)		
Swab (02)	2 (3.39%)	0 (0%)		

The association between the studied parameter and different clinical samples among Enterococcus isolates was analyzed using a total of 106 isolates. The samples included urine (n = 77), blood (n = 12), pus (n = 11), body fluids (n = 4), and swab samples (n = 2). Among these, urine samples showed 37 isolates (77.08%) positive, while 40 (68.97%) were negative. In blood samples, 5 isolates (10.42%) were positive, whereas 7 (12.07%) were negative. Pus samples demonstrated 4 isolates (8.33%) positive and 7 (12.07%) negative. Body fluid samples showed 1 isolate (2.08%) positive and 3 (5.17%) negative. Similarly, swab samples showed 1 isolate (2.08%) positive and 1 (1.72%) negative. Overall, 48 (45.28%) isolates were positive, while 58 (54.72%) were negative (Table 5).

**Table 5:** Distribution of gelE gene among different clinical sample types. Values are expressed as n (%). Percentages are column-wise. P-values were calculated using the chi-square test where appropriate. A p-value <0.05 was considered significant. The association between gelE gene presence and type of clinical sample was analyzed using the chi-square test and was found to be statistically non-significant (χ² = 1.34, p = 0.855; p > 0.05).

Sample	gelE gene positive 48 (45.28%)	gelE gene negative 58(54.72%)	Chi-square	P-value
Urine (77)	37 (77.08%)	40 (68.97%)	1.34	0.855
Blood (12)	5 (10.42%)	7 (12.07%)		
Pus (11)	4 (8.33%)	7 (12.07%)		
Body fluids (04)	1 (2.08%)	3 (5.17%)		
Swab (02)	1 (2.08%)	1 (1.72%)		

The distribution of the esp gene among different Enterococcus species was analyzed based on a total of 79 positive and 27 negative isolates (n = 106). Among the esp gene-positive isolates, *E. faecalis* and *E. faecium* each accounted for 30 cases (37.97%), followed by *E. durans* with 16 cases (20.25%) and *E. avium* with 3 cases (3.80%). In contrast, among the esp gene-negative isolates, *E. faecalis* represented the highest proportion with 13 cases (48.15%), followed by *E. faecium* with 10 cases (37.04%), *E. durans* with 3 cases (11.11%), and *E. avium* with 1 case (3.70%). Overall, when considering both positive and negative isolates together, *E. faecalis* was the most prevalent species, followed by *E. faecium*, while *E. durans* and *E. avium* contributed relatively fewer isolates (Table 6).

**Table 6:** Distribution of virulence gene esp among Enterococcus species. Values are expressed as n (%). Percentages are column-wise. P-values were calculated using the chi-square test where appropriate. A p-value <0.05 was considered significant. For the esp gene (chi-square = 1.45), a p-value of 0.69 was obtained, indicating no statistically significant difference in esp gene distribution across species (p > 0.05).

Species	esp gene positive (79)	esp gene negative (27)	Chi-square	P-value
Enterococcus faecalis	30 (37.97%)	13 (48.15%)	1.45	0.69
Enterococcus faecium	30 (37.97%)	10 (37.04%)		
Enterococcus durans	16 (20.25%)	3 (11.11%)		
Enterococcus avium	3 (03.80%)	1 (3.70%)		

The distribution of the cylA gene among different Enterococcus species was analyzed based on a total of 59 positive and 47 negative isolates (n = 106). Among the cylA gene-positive isolates, *E. faecium* accounted for the highest number of cases with 23 (38.98%), followed closely by *E. faecalis* with 22 cases (37.28%), *E. durans* with 11 cases (18.64%), and *E. avium* with 3 cases (5.08%). In contrast, among the cylA gene-negative isolates, *E. faecalis* represented the highest proportion with 21 cases (44.68%), followed by *E. faecium* with 17 cases (36.17%), *E. durans* with 8 cases (17.02%), and *E. avium* with 1 case (2.12%). Overall, when combining both positive and negative isolates, *E. faecalis* was the most prevalent species, followed by *E. faecium*, while *E. durans* and *E. avium* contributed comparatively fewer isolates (Table 7).

**Table 7:** Distribution of virulence gene cylA among Enterococcus species. Values are expressed as n (%). Percentages are column-wise. P-values were calculated using the chi-square test where appropriate. A p-value <0.05 was considered significant. For the cylA gene (chi-square = 1.05), a p-value of 0.79 was obtained, indicating no statistically significant difference in cylA gene distribution across species (p > 0.05).

Species	cylA gene positive (59)	cylA gene negative (47)	Chi-square	P-value
Enterococcus faecalis	22 (37.28%)	21 (44.68%)	1.05	0.79
Enterococcus faecium	23 (38.98%)	17 (36.17%)		
Enterococcus durans	11 (18.64%)	8 (17.02%)		
Enterococcus avium	3 (5.08%)	1 (2.12%)		

The distribution of the gelE gene among different Enterococcus species was analyzed based on a total of 48 positive and 58 negative isolates (n = 106). Among the gelE gene-positive isolates, *E. faecalis* accounted for the highest number of cases with 19 (39.58%), followed by *E. faecium* with 17 cases (35.41%), *E. durans* with 11 cases (22.91%), and *E. avium* with 1 case (2.08%). In contrast, among the gelE gene-negative isolates, *E. faecalis* again represented the highest proportion with 24 cases (41.37%), followed by *E. faecium* with 23 cases (39.65%), *E. durans* with 8 cases (13.79%), and *E. avium* with 3 cases (5.17%). Overall, when combining both positive and negative isolates, *E. faecalis* was the most prevalent species, followed by *E. faecium*, while *E. durans* and *E. avium* contributed comparatively fewer isolates (Table 8).

**Table 8:** Distribution of virulence gene gelE among Enterococcus species. Values are expressed as n (%). Percentages are column-wise. P-values were calculated using the chi-square test where appropriate. A p-value <0.05 was considered significant. For the gelE gene (chi-square = 2.03), a p-value of 0.57 was obtained, indicating no statistically significant difference in gelE gene distribution across species (p > 0.05).

Species	gelE gene positive (48)	gelE gene negative (58)	Chi-square	P-value
Enterococcus faecalis	19 (39.58%)	24 (41.37%)	2.03	0.57
Enterococcus faecium	17 (35.41%)	23 (39.65%)		
Enterococcus durans	11 (22.91%)	8 (13.79%)		
Enterococcus avium	1 (2.08%)	3 (5.17%)		

The association between virulence genes and vancomycin resistance among Enterococcus isolates was analyzed using a total of 106 isolates, categorized into vancomycin-sensitive (VSE) and vancomycin-resistant (VRE) groups. Among the esp gene-positive isolates (n = 79), 51 (64.55%) were vancomycin-sensitive, while 28 (35.44%) were vancomycin-resistant. In comparison, among the esp gene-negative isolates (n = 27), 18 (66.66%) were sensitive and 9 (33.33%) were resistant. Similarly, for the cylA gene, among positive isolates (n = 59), 38 (64.40%) were sensitive and 21 (35.59%) were resistant, whereas among negative isolates (n = 47), 31 (65.95%) were sensitive and 16 (34.04%) were resistant. In contrast, for the gelE gene, among positive isolates (n = 48), 24 (50%) were sensitive and 24 (50%) were resistant, while among negative isolates (n = 58), 45 (77.58%) were sensitive and 13 (22.41%) were resistant. Overall, these findings indicate that while esp and cylA genes do not show any significant relationship with vancomycin resistance, the gelE gene is significantly associated with resistance patterns among Enterococcus isolates (Table 9).

**Table 9:** Distribution of virulence genes (esp, cylA, and gelE) in relation to VRE antibiotic susceptibility (sensitive vs. resistant). Data are presented as counts (percentages). S: sensitive; R: resistant. Statistical analysis was performed using the Pearson chi-square test to evaluate the association between virulence gene positivity and vancomycin resistance (VRE 37). For the esp gene (chi-square = 0.04), a p-value of 0.84 was obtained, indicating no statistically significant association between the esp gene and antibiotic resistance pattern (p > 0.05). For the cylA gene (chi-square = 0.03), a p-value of 0.87 was obtained, indicating no statistically significant association between the cylA gene and antibiotic resistance pattern (p > 0.05). For the gelE gene (chi-square = 8.79), a p-value of 0.001 was obtained, indicating a statistically significant association between the gelE gene and antibiotic resistance pattern (p < 0.05).

Genes	Groups	VRE 37		Chi-square	p-value
		S	R		
esp gene	Positive (79)	51 (64.55%)	28 (35.44%)	0.04	0.84
	Negative (27)	18 (66.66%)	9 (33.33%)		
cylA gene	Positive (59)	38 (64.40%)	21 (35.59%)	0.03	0.87
	Negative (47)	31 (65.95%)	16 (34.04%)		
gelE gene	Positive (48)	24 (50%)	24 (50%)	8.79	0.001
	Negative (58)	45 (77.58%)	13 (22.41%)		

The relationship between antimicrobial susceptibility patterns and virulence genes (esp, cylA, and gelE) was analyzed among 106 Enterococcus isolates. For the esp gene (n = 106; positive = 79, negative = 27), the highest sensitivity was observed for linezolid (71/79 (89.8%)) and vancomycin (51/79 (64.5%)), whereas the highest resistance was noted for erythromycin (75/79 (94.9%)). Statistically significant associations were observed between the esp gene and penicillin (χ² = 6.87, p = 0.009), chloramphenicol (χ² = 5.25, p = 0.022), erythromycin (χ² = 4.69, p = 0.030), and nitrofurantoin (χ² = 7.21, p = 0.007). For the cylA gene (positive = 59, negative = 47), isolates showed high sensitivity to linezolid (52/59 (88.1%)) and vancomycin (38/59 (64.4%)). High resistance was observed for erythromycin (53/59 (89.8%)), penicillin (49/59 (83.0%)), and tetracycline (48/59 (81.3%)). However, no statistically significant association was found between the cylA gene and antimicrobial susceptibility (p > 0.05). For the gelE gene (positive = 48, negative = 58), the highest sensitivity was observed for linezolid (43/48 (89.6%)) and moderate sensitivity for vancomycin (24/48 (50.0%)). High resistance was observed for erythromycin (45/48 (93.7%)) and tetracycline (41/48 (85.4%)). A statistically significant association was observed for high-level gentamicin (χ² = 4.49, p = 0.034) and vancomycin (χ² = 8.80, p = 0.003). Overall, linezolid was the most effective antibiotic across all virulence gene groups, while erythromycin exhibited the highest resistance. Statistical significance was considered at p < 0.05 (Table 10).

**Table 10:** Association of virulence genes (esp, cylA, and gelE) with antimicrobial susceptibility patterns among Enterococcus isolates (n=106). Values are expressed as n (%). Percentages are column-wise. P-values were calculated using the chi-square test where appropriate. A p-value <0.05 was considered significant. S: sensitive; R: resistant. Data are presented as a count (percentage). χ²: chi-square value; p: p-value. *n=77 for norfloxacin and nitrofurantoin. Statistical analysis was performed using the Pearson chi-square test to evaluate the association between virulence gene positivity and antimicrobial susceptibility. Highlighted p-values indicate statistically significant associations (p < 0.05). For the esp gene: significant associations were observed with penicillin (p=0.009), chloramphenicol (p=0.022), erythromycin (p=0.030), nitrofurantoin (p=0.007), high-level gentamicin (p=0.034), and vancomycin (p=0.003); all other antimicrobials showed no significant association (p > 0.05). For the cylA gene: no statistically significant associations were observed with any of the tested antimicrobial agents (all p > 0.05). For the gelE gene: significant associations were observed with high-level gentamicin (p=0.034) and vancomycin (p=0.003); all other antimicrobials showed no significant association (p > 0.05).

Antimicrobial agent	S/R	esp gene (n=106)				cylA gene (n=106)				gelE gene (n=106)			
		Positive 79 (%)	Negative 27 (%)	esp: χ²	esp: p	Positive 59 (%)	Negative 47 (%)	cylA: χ²	cylA: p	Positive 48 (%)	Negative 58 (%)	gelE: χ²	gelE: p
High-level gentamicin (120 µg)	S	28 (35.4)	17 (40.7)	0.02	0.88	18 (30.5)	20 (42.5)	1.65	0.20	12 (25.0)	26 (44.8)	4.49	0.034
	R	51 (64.5)	17 (62.9)			41 (69.4)	27 (57.4)			36 (75.0)	32 (55.1)		
Vancomycin (30 µg)	S	51 (64.5)	18 (66.6)	0.04	0.84	38 (64.4)	31 (65.9)	0.03	0.87	24 (50.0)	45 (77.5)	8.80	0.003
	R	28 (35.4)	9 (33.3)			21 (35.5)	16 (34.0)			24 (50.0)	13 (22.4)		
Linezolid (15 µg)	S	71 (89.8)	21 (77.7)	2.57	0.11	52 (88.1)	40 (85.1)	0.21	0.65	43 (89.5)	49 (84.4)	0.60	0.44
	R	8 (10.1)	6 (22.2)			7 (11.8)	7 (14.8)			5 (10.4)	9 (15.5)		
Doxycycline (30 µg)	S	20 (25.3)	9 (33.3)	0.65	0.42	13 (22.0)	16 (34.0)	1.89	0.17	11 (22.9)	18 (31.0)	0.87	0.35
	R	59 (74.6)	18 (66.6)			46 (77.9)	31 (65.9)			37 (77.0)	40 (68.9)		
Penicillin (10 units)	S	9 (11.3)	9 (33.3)	6.87	0.009	10 (16.9)	8 (17.0)	0.00	1.00	7 (14.5)	11 (18.9)	0.36	0.55
	R	70 (88.6)	18 (66.6)			49 (83.0)	39 (82.9)			41 (85.4)	47 (81.0)		
Ciprofloxacin (5 µg)	S	22 (27.8)	12 (44.4)	2.54	0.11	21 (35.5)	13 (27.6)	0.76	0.38	17 (35.4)	17 (29.3)	0.45	0.50
	R	57 (72.1)	15 (55.5)			38 (64.4)	34 (72.3)			31 (64.5)	43 (74.1)		
Chloramphenicol (30 µg)	S	27 (34.1)	16 (59.2)	5.25	0.022	25 (42.3)	18 (38.2)	0.18	0.67	20 (41.6)	23 (39.6)	0.04	0.83
	R	52 (65.8)	11 (40.7)			34 (57.6)	29 (61.7)			28 (58.3)	35 (60.3)		
Erythromycin (15 µg)	S	4 (5.0)	5 (18.5)	4.69	0.030	6 (10.1)	3 (6.3)	0.48	0.49	3 (6.2)	6 (10.3)	0.57	0.45
	R	75 (94.5)	22 (81.4)			53 (89.8)	44 (93.6)			45 (93.7)	52 (89.6)		
Tetracycline (30 µg)	S	11 (13.9)	7 (25.9)	2.61	0.11	10 (16.9)	8 (17.0)	0.03	0.87	6 (12.5)	12 (20.6)	1.25	0.26
	R	66 (83.5)	20 (74.0)			48 (81.3)	38 (80.8)			41 (85.4)	45 (77.5)		
Norfloxacin* (10 µg)	S	2 (3.3)	1 (3.7)	1.91	0.17	2 (4.8)	1 (2.7)	0.89	0.34	1 (2.7)	2 (5.0)	1.14	0.29
	R	58 (96.6)	16 (94.1)			39 (95.1)	36 (97.2)			36 (97.2)	38 (95.0)		
Nitrofurantoin* (300 µg)	S	19 (31.6)	11 (64.7)	7.21	0.007	16 (39.0)	14 (38.8)	0.67	0.41	14 (50.0)	15 (37.5)	0.95	0.33
	R	41 (68.3)	6 (35.2)			25 (60.9)	22 (61.1)			22 (59.4)	25 (62.5)		

## Discussion

In our study, Enterococcus strains were tested for virulence genes by the reverse transcription polymerase chain reaction (RT-PCR) method for molecular detection, such as the esp gene, cylA gene, and gelE gene. Out of 106 enterococcus isolates, 74.53% show positive esp gene, followed by 55.66% showing positive cylA gene, and 45.28% show gelE gene, respectively. According to similar findings by Weng et al. [14], human infection severity is significantly influenced by cytolysin. In another finding, phenotypic hemolytic activity was found in 30.8% of *E. faecalis*, whereas 43.5% had cylA. In support of the results of this investigation. In another study by Jahansepas et al. [15] demonstrated that 41% of Enterococci have the cylA gene, and hemolytic activity was found in 38% of cylA-positive isolates. The study's multiplex PCR procedure, which simultaneously detected five distinct virulence genes, turned out to be a dependable and quick substitute for uniplex PCR and phenotypic testing [16]. 

According to this, the main virulence factor is gelatinase. Virulence factor from catheter-induced infection, blood, and wounds. The presence of quiet genes may be the reason why more strains tested positive for the gelE gene by PCR than strains that tested positive in phenotypic assays. 

For a more comprehensive characterization of the strains, both phenotypic and genotypic analyses are essential. No single gene was found to be significantly associated with biofilm formation, suggesting that this process is multifactorial and depends on the interaction of multiple genes along with various environmental factors [8].

According to Strateva et al. [17], the cytolysin structural gene was never absent from β-hemolytic *E. faecalis*, although it was absent from *E. faecium*. Therefore, another cytotoxic factor must be responsible for the hemolysis of *E. fecium*. Similar to the results of this investigation in *E. faecium* isolates, 75 isolates with hemolytic activity in another study tested negative for the cylA gene, indicating a potential involvement of additional genes in hemolytic activity. By encouraging cell-to-cell contact and the conjugal transfer of plasmids carrying antibiotic resistance and virulence, the fecal VRE's capacity to create slime is especially crucial for resistance acquisition genes. These strains may aid in the spread of these bacteria in hospital environments and seem to be the entrance point for novel resistance genes into enterococcal species in the gastrointestinal tract [17,18].

According to our results, most esp virulence gene-positive and negative strains show sensitivity and resistance to antibiotics. Highly sensitive to linezolid and vancomycin (89.8% and 64.5%) and highly resistance in erythromycin 94.4% shown in esp gene because other study by Armin et al. [19] the presence of esp was higher in clinical infections and isolates linked to epidemics than in surveillance isolates, research revealed that the variant esp gene in vancomycin-resistant *E. faecium* and vancomycin-sensitive *E. faecium* was strongly associated with a particular epidemiologic source [20].

The most resistant antibiotic was ciprofloxacin. Only 5.21% of cases had vancomycin-resistant enterococci (VRE), and the majority of these cases had Van A-type VRE detected phenotypically. By encouraging cell-to-cell contact and the conjugal transfer of plasmids carrying antibiotic resistance and virulence, the fecal VRE's capacity to create slime is especially crucial for resistance acquisition genes. These strains may aid in the spread of these bacteria in hospital environments and seem to be the entrance point for novel resistance genes into enterococcal species in the gastrointestinal tract [6,21].

In addition, the prevalence of multidrug-resistant (MDR) Enterococcus spp., including vancomycin-resistant enterococci (VRE), has been reported to be increasing in India, posing a significant clinical challenge. This rising resistance trend enhances the ability of enterococci to survive under antimicrobial pressure and increases the likelihood of interactions with other drug-resistant pathogens.

Limitations of the study

This study has certain limitations that should be considered while interpreting the results. The sample size was relatively small, and the study was conducted at a single center, which may limit the generalizability of the findings. Only selected virulence genes (cylA, gelE, and esp) were analyzed, and other potentially relevant genes were not included. Additionally, advanced molecular techniques and detailed genetic characterization were not performed. Furthermore, clinical outcomes and long-term follow-up data were not assessed. Future studies with larger sample sizes, multi-center involvement, and comprehensive molecular analysis are recommended.

## Conclusions

This study evaluated the prevalence, antibiotic susceptibility, and clinical significance of enterococcal infections in a hospital setting. *Enterococcus faecalis* and *Enterococcus faecium* were the most common isolates, but the emergence of less common species highlights the need for accurate species-level identification and further research into regional prevalence. These findings emphasize the importance of cautious use of critical antibiotics, such as vancomycin and linezolid, along with strict infection control measures to prevent the spread of resistant strains like VRE. Continuous monitoring of antibiotic susceptibility patterns, regular screening, and minimum inhibitory concentration (MIC) testing are essential for guiding effective treatment strategies and developing regional antibiotic policies.
